# Single-cell specific and interpretable machine learning models for sparse scChIP-seq data imputation

**DOI:** 10.1371/journal.pone.0270043

**Published:** 2022-07-01

**Authors:** Steffen Albrecht, Tommaso Andreani, Miguel A. Andrade-Navarro, Jean Fred Fontaine

**Affiliations:** 1 Institute of Organismic and Molecular Evolution (iOME), Faculty of Biology, Johannes Gutenberg University Mainz, Mainz, Germany; 2 Institute of Molecular Biology, Mainz, Germany; Pohang University of Science and Technology, REPUBLIC OF KOREA

## Abstract

**Motivation:**

Single-cell Chromatin ImmunoPrecipitation DNA-Sequencing (scChIP-seq) analysis is challenging due to data sparsity. High degree of sparsity in biological high-throughput single-cell data is generally handled with imputation methods that complete the data, but specific methods for scChIP-seq are lacking. We present SIMPA, a scChIP-seq data imputation method leveraging predictive information within bulk data from the ENCODE project to impute missing protein-DNA interacting regions of target histone marks or transcription factors.

**Results:**

Imputations using machine learning models trained for each single cell, each ChIP protein target, and each genomic region accurately preserve cell type clustering and improve pathway-related gene identification on real human data. Results on bulk data simulating single cells show that the imputations are single-cell specific as the imputed profiles are closer to the simulated cell than to other cells related to the same ChIP protein target and the same cell type. Simulations also show that 100 input genomic regions are already enough to train single-cell specific models for the imputation of thousands of undetected regions. Furthermore, SIMPA enables the interpretation of machine learning models by revealing interaction sites of a given single cell that are most important for the imputation model trained for a specific genomic region. The corresponding feature importance values derived from promoter-interaction profiles of H3K4me3, an activating histone mark, highly correlate with co-expression of genes that are present within the cell-type specific pathways in 2 real human and mouse datasets. The SIMPA’s interpretable imputation method allows users to gain a deep understanding of individual cells and, consequently, of sparse scChIP-seq datasets.

**Availability and implementation:**

Our interpretable imputation algorithm was implemented in Python and is available at https://github.com/salbrec/SIMPA.

## Introduction

The discovery of protein-DNA interactions of histone marks and transcription factors is of great importance in biomedical studies because of their impact on the regulation of core cellular processes such as chromatin structure organization and gene expression. These interactions are measured by chromatin immunoprecipitation followed by high-throughput sequencing (ChIP-seq). Public data from the ENCODE portal, which provides a large collection of experimental bulk ChIP-seq data, has been used for comprehensive investigations providing insights into epigenomic processes that affect chromatin 3D-structure, chromatin state, and gene expression, to name just a few [1, 2].

Recently developed protocols for scChIP-seq are powerful techniques that will enable in-depth characterization of those processes at single-cell resolution. ChIP-seq was successfully performed on single cells with sequencing depth as low as 1,000 unique reads per cell, reflecting the low amount of cellular material that can be obtained from only one single cell [3]. Even though this low coverage leads to sparse datasets, scChIP-seq data has enabled the study of biological systems that cannot be investigated with bulk ChIP-seq applied for many cells, for example, the differences between drug-sensitive and drug-resistant breast cancer cells [4].

The analysis of single-cell assays is strongly affected by the sparsity of the data. In the context of ChIP-seq, sparsity means no signal observed for numerous genomic regions without the possibility to explain whether this is real or due to low sequencing coverage. Notably, sparsity may disable the investigation of functional genomic elements that could be of crucial interest. Hence, an imputation method is needed to complete sparse scChIP-seq datasets while preserving the identity of each individual cell.

The first published imputation method for bulk NGS epigenomic signals was ChromImpute [5], later followed by [6], an improved method for the imputation of signal tracks for several molecular assays in a biosample-specific manner (*biosample* refers to the specific tissue or cell-type, not to single cell). The challenge of transcription factor binding site prediction was approached, for example, using deep learning algorithms on sequence position weight matrices [7] and more recently by the embedding of transcription factor labels and k-mers [8]. With the aim to complete the ENCODE portal with imputed bulk experiments, Schreiber *et al*. implemented the method Avocado, which extends the basic concept of PREDICTD by deep neural networks [9]. Avocado was also validated on ChIP-seq data from both histone marks and transcription factors [10]. Such methods show the successful application of machine learning algorithms and mathematical approaches in predicting epigenomic signals such as transcription factor binding activity. However, their scope, being limited to either imputation of missing bulk experiments or sequence-specific binding site prediction, hampers their application to single-cell data.

The challenge of imputation for sparse datasets from single-cell assays has been extensively approached for single-cell RNA-seq (scRNA-seq) used to quantify gene expression at single-cell resolution e.g. [11–19]. In this context, similarly to scChIP-seq data, sparsity is described by dropout events, which are transcripts having a transcription rate of zero without knowing if the corresponding gene is not expressed at all or if the expression rate is not detected due to technical limitations [16]. The question arises if these methods can be easily adapted for imputation of scChIP-seq data. However, there are crucial differences between the application of RNA-seq and ChIP-seq techniques that must be considered regarding the development of a method for scChIP-seq imputation.

First, in RNA-seq the set of relevant genomic regions, defined by the species-specific transcripts, is more limited. For a ChIP-seq profile, the regions of potential interest may originate from any position in the genome and cannot be defined in advance. To simplify the analysis, in scChIP-seq imputation the genome can be organized in non-overlapping genomic windows (bins) of a certain size. At 5 kb resolution, this binning concept results in more than 600,000 possible bins in the human genome, a number that is much higher than the number of transcripts considered in the RNA-seq context.

The second main difference is that scChIP-seq interactions are usually represented by a Boolean value (the value can be either “True” or “False”) describing the presence or absence of a significant enrichment of sequencing reads defining a peak, while RNA-seq datasets contain transcription rates. Consequently, the application of scRNA-seq imputation methods on scChIP-seq data might be less appropriate.

In contrast, imputation methods for chromatin accessibility profiles from single-cell ATAC-seq (single-cell Assay for Transposase-Accessible Chromatin using sequencing, scATAC-seq) are potentially more transferable to scChIP-seq imputation as their data representation is more similar. A few methods exist that implement imputation for scATAC-seq, though none of them was tested on scChIP-seq data so far. Methods such as SCALE which uses a combination of Gaussian mixture models and variational autoencoder [20] have been shown to outperform scRNA-seq methods to impute scATAC-seq data (see also FITs [21] and scOpen [22]). These methods complement each other with respect to the different approaches they implement, however, they share the common concept of imputing the missing values within a sparse matrix defined by the single cells (rows) and the genomic bins (columns) of a given experiment, and only bins are considered that were detected by at least one single cell if no further filtering is applied. Consequently, such methods can offer imputation only on regions that were observed in the single-cell dataset and it is likely that many important regions along the whole genome will be missed.

To overcome this limitation, we developed SIMPA, an algorithm for **S**ingle-cell Ch**I**P-seq i**MP**ut**A**tion, that uses bulk ChIP-seq datasets of the ENCODE project to train its machine learning models [2, 23]. It was already shown that an additional bulk RNA-seq dataset can be used to improve the imputation for a sparse scRNA-seq dataset [14]. Within SIMPA, publicly available bulk ChIP-seq data is turned into a reference set used to define potential bins to be imputed. Bins observed in the single-cell dataset are then used to derive training sets specified by both the bulk reference data and single-cell data. This training set is then leveraged by machine learning models to compute specific imputation probabilities. Moreover, these models are interpretable and can be used to gain insights into a given single-cell dataset, allowing a more detailed investigation of individual cells. The interpretability is implemented by InterSIMPA, an extension which takes a single cell as input together with a genomic position of interest and trains one classification model for the position to derive a probability which can be seen as an imputation score. More importantly, InterSIMPA ranks the genomic bins from the sparse single-cell profile by their relevance for the model. The ranked bins are enriched by detailed information and an importance score describing the strength of their relationship with the given genomic position of interest. These relationships can be interpreted as dependencies between genomic regions that could be part of the gene regulatory network (e.g., between enhancers and promoters).

The basic reference-based imputation concept of SIMPA was first validated for human on simulated data and then on a real scChIP-seq dataset of immune cells [4]. The simulated data was used to demonstrate the single-cell specificity of the imputations on full data profiles. The real dataset allowed us to investigate the algorithm’s capability of retaining the cell-type clustering and furthermore to assess the biological relevance of the imputed regions based on a pathway enrichment analysis. Results from InterSIMPA were validated using promoter regions related to genes of the B- and T-cell signaling pathways in the human dataset and regions related to brain functions in a mouse dataset [24].

## Methods

### Datasets

#### Preparation of the reference data (bulk ChIP-seq datasets)

To create the reference set that is used by SIMPA we downloaded all ChIP-seq experiments from the ENCODE portal that comply with the following criteria: the status is released, the experiment is replicated (isogenic or anisogenic), no treatment to the biosample, without genetic modification, and the organism is *Homo Sapiens* (human) or *Mus Musculus* (mouse). Experiments represent different ChIP protein targets (antibody targets within the ChIP) and biosamples (either tissue or an immortalized cell line). For all experiments, we downloaded fully preprocessed sets of protein-DNA interacting regions as peak files: the *replicated peaks* for histone mark ChIP and the *optimal IDR thresholded peaks* for transcription factor ChIP. For human, 2251 peak files of the hg19 or hg38 assembly were downloaded and preprocessed as hg38 after converting hg19 files with the UCSC LiftOver tool [25]. For mouse, 848 peak files of the mm10 assembly were downloaded and preprocessed (data from other assemblies did not complement the selection). For downloading, preprocessing, and updating the datasets we used a semi-automatic, SQL-backend procedure that was already used in a previous project [26]. A list with all 3099 reference experiments is provided in S1 Table including information about the protein target, the cell-type or tissue, the assembly, and the exact ENCODE accession IDs.

#### Data preprocessing

In order to limit computational complexity, all reference experiments were converted from ChIP-seq peak sets to genomic bin sets (bins are defined as non-overlapping regions on a genome). We provide on github the reference data in bin sizes (or resolution) of 5 kb and 50 kb for hg38 (https://github.com/salbrec/simpa). We also provide the reference data for mm10 for some of the main histone marks in different sizes. However, the github repository also provides scripts and descriptions that enable the preparation of any target in the desired resolution (bin sizes). Bin sets for the reference ChIP-seq experiment are in binary format to be more efficiently integrated by the main Python scripts. Given one reference experiment, a bin is said to be “present” if there is at least one ChIP-seq peak that overlaps this bin, “absent” otherwise.

#### Preprocessing of scChIP-seq data for human (Grosselin *et al*., 2019)

We downloaded the count matrices for H3K4me3 and H3K27me3 available in GEO under accession number GSE117309 in 5 kb and 50 kb binning resolution, respectively. From the matrices, we derived bed files for every single cell excluding gender-specific chromosomes. SIMPA, InterSIMPA and other imputation methods were then applied on 25% of the single cells randomly sampled, 1520 bed files for H3K4me3 and 1128 bed files for H3K27me3.

#### Preprocessing of scChIP-seq data for mouse (Zhu et al., 2021)

We downloaded the count matrices for H3K4me3 available in GEO under accession number GSE152020 in 1kb binning resolution. From the matrices, we derived bed files for every single cell excluding gender-specific chromosomes. The initial dataset provides 7465 cells for this histone mark; we applied InterSIMPA on a subset of 1000 randomly sampled cells.

### The SIMPA algorithm

**Fig 1:**
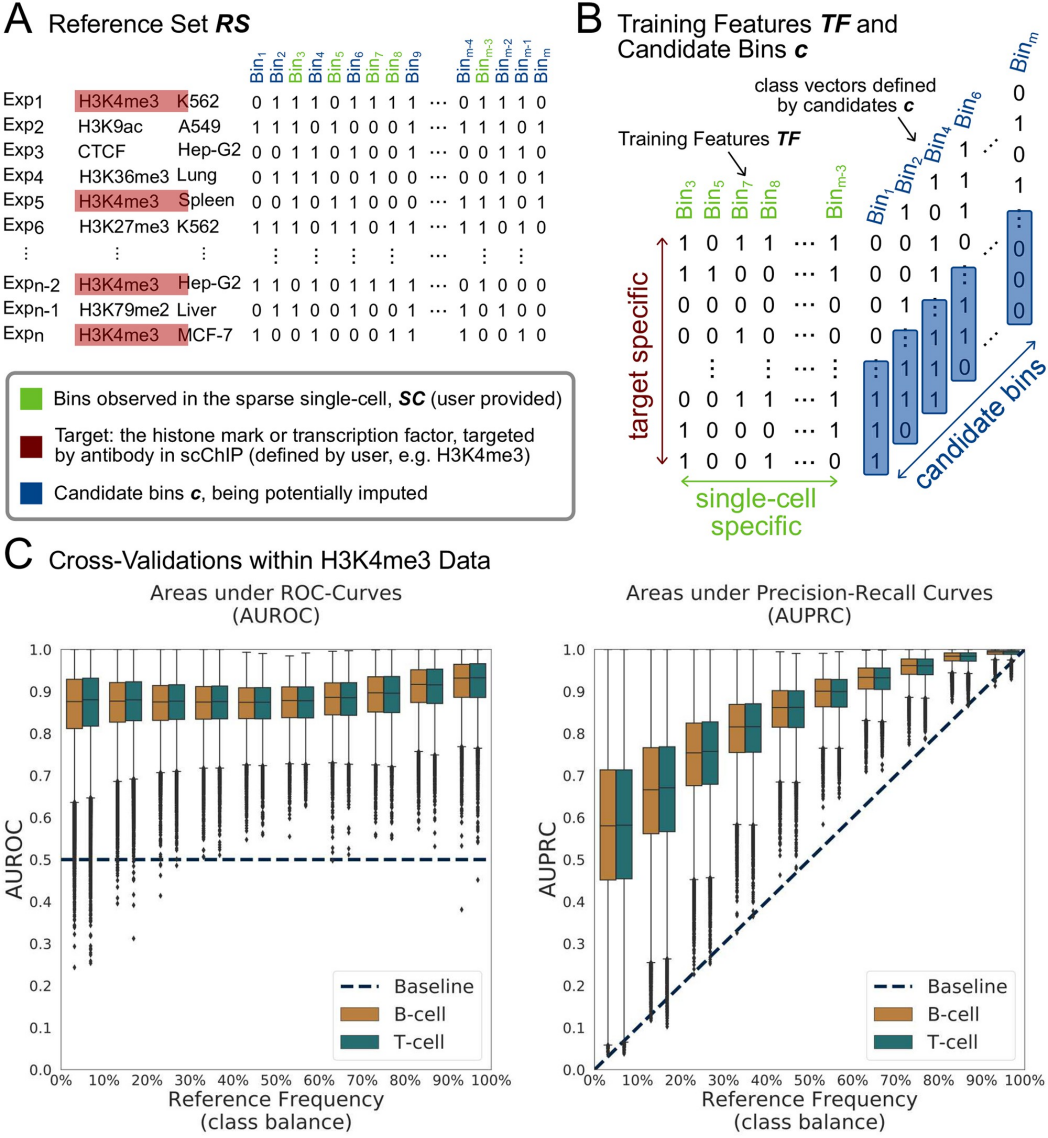
SIMPA’s algorithm and cross validations. **A**. Identified ChIP-seq regions from bulk experiments were downloaded from ENCODE and mapped to bins defined as non-overlapping and contiguous genomic regions of a defined length (5 kb for H3K4me3 and 50 kb for H3K27me3 in the human dataset) and covering the whole genome (the table). A bin is given a value of 1 for a particular experiment if there is at least one ChIP-seq region in this experiment that overlaps the bin, 0 otherwise. In total 2251 human and 848 mouse ChIP-seq experiments for several targets (histone marks or transcription factors) performed in several biosamples (tissues and cell-lines) were downloaded from ENCODE portal and preprocessed. Depending on the target specified by the user, the target-specific reference set **RS** is then created and contains all experiments related to this target (rows in red; H3K4me3 is given as example) and all bins observed for at least one of those experiments. **B**. The single-cell specific training feature matrix **TF** is created as a subset of **RS** by selecting only bins observed within the given single cell (green columns). All other bins from **RS** are the candidate bins (**c**; blue columns) and define the class vectors consisting of the corresponding values in **RS**. For each candidate bin, a classification model is trained based on the training features and the class vector identifying associated experiments. **C**. Cross-validated evaluations of SIMPA’s Random Forest performances to predict values of candidate bins defined for real human single-cell data related to H3K4me3. For each candidate bin, a ten-fold cross-validation was applied and summarized as the mean Area under ROC-Curve (AUROC) or Area under Precision-Recall Curve (AUPRC) (y-axes). Results for all bins are represented by boxplots subdivided by class balance in the candidate bins (percentage of “1” values in the bin) (x-axis). The dashed lines describe the baseline performance expected from a random classification model: 0.5 for AUROC and equal to the class balance for AUPRC.

SIMPA is an algorithm implemented in Python 3.7.3 for “**S**ingle-cell Ch**I**P-seq i**MP**ut**A**tion”, which is applied to one single cell represented by a sparse set of scChIP-seq genomic regions (or peaks) provided by the user in bed format. Within the algorithm, the given single-cell bed file is converted into a set of bins *SC* describing the single-cell input (Fig 1A). The user also provides the protein target name for the histone mark or transcription factor targeted by the antibody within the single-cell immunoprecipitation. The target name is needed to specify the training set, which consists of experiments from the ENCODE reference set.

Given the sparse profile of one single cell as input, SIMPA aims to impute missing bins based on predictive information within bulk data specified by the selected ChIP protein target and further by the regions taken from the input single-cell profile. In order to make the bulk data informative, first SIMPA collects all the ENCODE reference experiments available for the given target that defines the rows of the reference set matrix (*RS*) where columns represent bins (Fig 1A):

$$RS=\left(a_{i,j}\right),\;1\leq i\leq n,\;1\leq j\leq m$$

*with*

$$a_{i,j}\in \left\{0,1\right\}\;\text{describing}\;\text{a}\;\text{cell}\;\text{of}\;\text{the}\;\text{matrix}\;\text{with}\;\text{value}=1\;\text{when}\;\text{bin}\;j\;\text{in}\;\text{reference}\;\text{experiment}\;i\;\text{is}\;\text{present},\;0\;\text{otherwise},$$

and where *n* is the number of experiments available for the given target, and *m* is the number of bins that are present in at least one of the target specific experiments. As the rows are defined by the given target, the target-specificity is induced within this step.

Second, a subset of *RS* is created by selecting only the columns for bins that are present in the given single-cell profile *SC* to create the training features *TF*:

TF⊂RS,

$$TF=\left(a_{i,k}\right),\;1\leq i\leq n,\;1\leq k\leq s,$$

where *k* indexes a selection of bins from *RS* that are present in *SC* and with *s* the number of bins in *SC* (Fig 1B). Bins present in *RS* but not in *SC* are collected and named as candidate bins *c* that are potentially imputed bins.

Third, SIMPA takes each candidate bin *c*_*g*_ in *c* (*c* is the set of candidate bins; the number of bins in *c* is *P*; thus 1 ≤ g ≤ P) separately to compute an individual imputed probability *ρ*_*g*_ that *c*_*g*_ is present in the single cell. Given *c*_*g*_, SIMPA trains a classification model *cm*_*g*_ based on *TF* defining the features and *c*_*g*_ the class vector. Because an individual model is trained for each individual candidate bin, bin-specificity is induced for the whole approach. The imputed probability *ρ*_*g*_ is finally computed by *cm*_*g*_ which takes as input an artificial instance vector *b* = (*b*_*k*_), *b*_*k*_ = 1, 1 ≤ *k* ≤ *s*. Consequently, *ρ*_*g*_ is the probability of *c*_*g*_ to be predicted for the imputed single-cell result, given the fact that all bins in *SC* are observed. As we use a Random Forest implementation from the scikit-learn (version 0.21.3) Python’s library [27, 28] with default settings to build classification models, the imputed probability is then the mean predicted class probability of the trees (by default 100) in the forest while the class probability of a single tree is calculated by the fraction of samples of the same class in a leaf.

When applying this algorithmic strategy on a real sparse single-cell profile to impute candidate bins, the final step of receiving the imputed probability differs from any cross-validation scenario as there is no hold-out sample that could be used to apply the model on. Instead, the model is applied on a synthetic vector containing only 1s to receive the imputed probability. Providing this vector in which the interaction is present for each bin, differs to the nature of the reference data which usually describes a mixture of interaction and non-interaction. However, this strategy is applied in the same way for all candidate bins excluding a potential bias regarding the ranking of candidate bins by the imputed probability. More crucially, by this final step we force the Random Forest model to return an imputed probability based on the knowledge that all bins captured by a single cell are present, thus, the outcome is highly specific to the given single cell. Moreover, this strategy allows us to keep focus on a cell while interpreting the underlying model to gain more insights with high specificity to the given individually single cell.

Finally, SIMPA creates two files: one file in bed format and the other in SIMPA format described as a table listing the single-cell bins first, followed by the imputed bins sorted by the imputed probability. A line represents a bin described by its ID, its genomic coordinates, its frequency according to the target-specific reference experiments, and the imputed probability. Note that the first bins on top of this file have no imputed probabilities as they represent the original sparse single-cell input (a default value of -1 is assigned). The second file created by SIMPA is the imputed bed file containing the original single-cell bins and the additional imputed bins selected among those with the highest imputed probability. The number of bins within this bed file is defined by the average number of bins present in the target-specific bulk experiments, e.g. 32,584 for H3K4me3 (5 kb bin size) and 12,598 for H3K27me3 (50 kb bin size) on the hg38 samples.

### Cross validations

Stratified ten-fold cross-validations were done to verify if the Random Forest Classifier used by SIMPA when applied to the real single-cell dataset is able to make use of statistical patterns from the bulk data to train accurate models predicting the presence or absence of a protein-DNA interaction in candidate bins. Hence, within this analysis, the performance of Random Forest was cross-validated on the candidate bin values not used in the training set but still defined in the reference set. We chose Random Forest as, by default, the algorithm never uses all given features for training one decision tree and consequently smaller sets of genomic bins are considered for a tree which agrees with the biological assumption that not all regions, captured by an individual single cell, are relevant for the imputation of a candidate bin. Given a candidate bin, SIMPA trains a Random Forest with 100 decision trees (number of estimators) and aggregating the votes from all trees results in a probability we use as imputed probability.

We applied the following cross-validation approach (results shown in Fig 1C and Fig S1 in S1 File): given a protein target (either H3K4me3 or H3K27me3), 10 single cells were randomly chosen from the real dataset for each cell type; for each single cell the training feature matrix TF for SIMPA was created as explained above together with the set of candidate bins. Then, for each candidate bin that defines the class vector, a Random Forest classification model was trained and evaluated by the area under the ROC-curve within a stratified ten-fold cross-validation. In addition, we used the area under precision-recall curve to better study the class vector imbalance.

## Results

### Algorithmic concept and cross-validations

Unlike many other single-cell imputation methods, SIMPA leverages predictive information within bulk ChIP-seq data by combining the sparse input of one single cell and a collection of bulk ChIP-seq experiments from ENCODE. In order to better compare bulk and single-cell data, ChIP-seq regions (or significant signal/noise ChIP-seq peaks) are mapped to genomic bins (Fig 1A; see Methods **for details about bulk and single-cell data retrieval and processing**).

SIMPA produces results for each single cell of a scChIP-seq dataset by using machine learning models trained on a subset of the ENCODE data related to a selected ChIP protein target, that is the histone mark or transcription factor used in the single-cell experiment. Derived from this target-specific subset, the classification features are defined by genomic bins detected in the single cell, while the class to predict is defined by a bin observed in at least one target-specific bulk ENCODE experiment, but not in the single cell (Fig 1B). In other words, by using this particular data selection strategy, SIMPA searches relevant statistical patterns linking (i) the bulk ChIP-seq data across single-cell-related bins and target-related experiments for different cell types to (ii) the potential presence or absence of a bin in the given single cell.

Within a cross-validation scenario that compared the predicted probabilities to corresponding candidate bin values, results show that the machine learning-concept of SIMPA is able to provide accurate predictions (Fig 1C, **Supplementary Note 1 in** S1 File, and **Fig S1 in** S1 File). Moreover, on the high-resolution H3K4me3 human dataset [4], SIMPA achieved high recall rates for bins removed from single-cell profiles (**Supplementary Note 2** and **Fig S2 in** S1 File).

### Validation on simulated data

In order to evaluate the algorithm’s ability from few input bins (hundreds) to complete full data profiles (thousands of bins) of different protein targets and cell-types, we simulated sparse protein-DNA interaction profiles from the bulk ENCODE ChIP-seq experiments that are used as reference data by SIMPA. For the simulation, we took human bulk experiments for different cell-type-target combinations to define them as full single-cell profiles (*origin*) and down-sampled those profiles to simulate sparse single-cell profiles (from 100 to 1600 randomly selected bins) (see **Supplementary Note 3 in** S1 File for details). Each simulated sparse profile was used as input for SIMPA and the output was compared to the origin (excluding bins used as input). For the model training, the full origin profile was excluded from the reference training set in order to apply the default validation, called *leave-out origin* (LOO). Additionally, a more challenging validation strategy was applied in which all reference profiles for the same cell-type (biosample) were excluded, called *leave-out cell-type* (LOCT).

**Fig 2:**
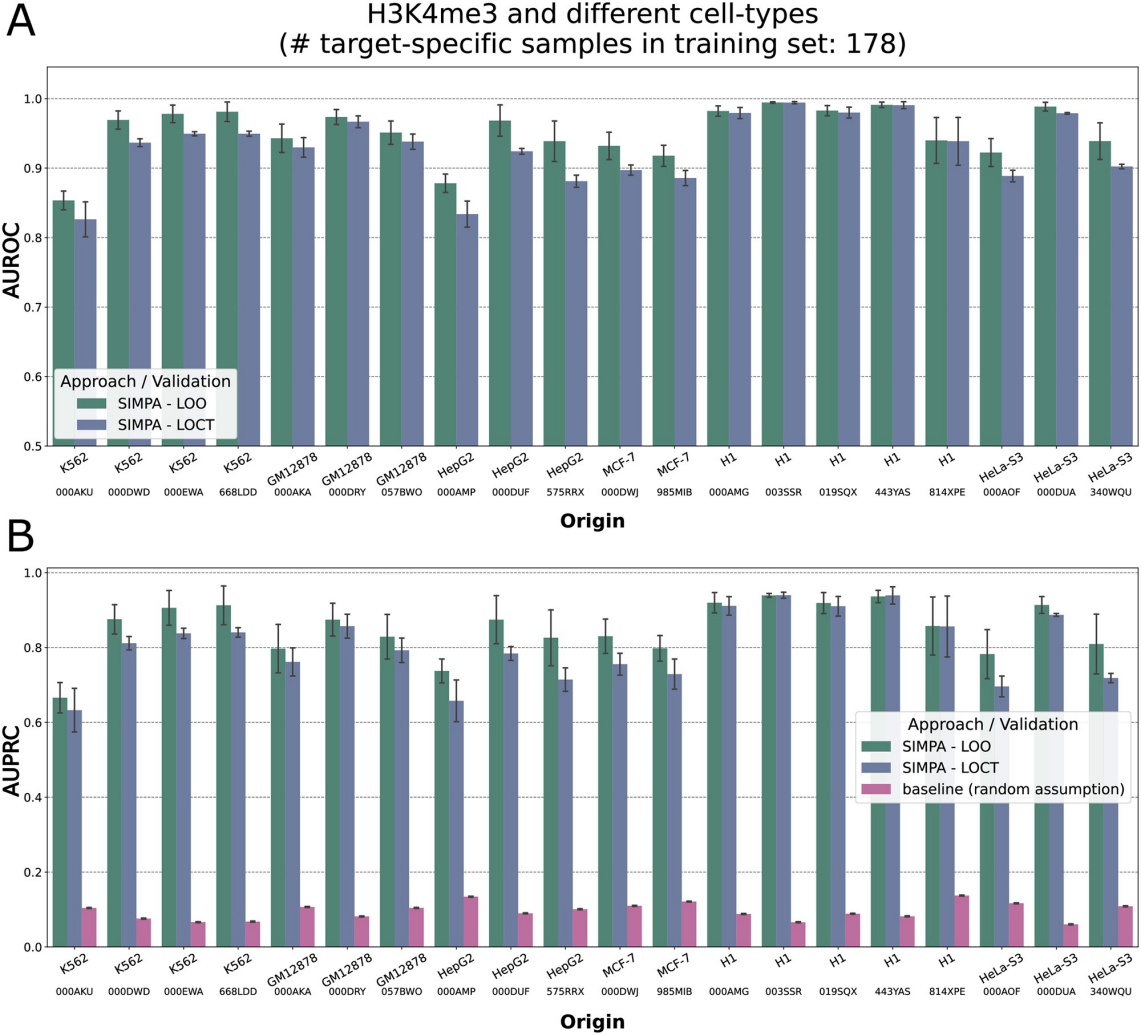
Performance on simulated sparse profiles in different cell-types. **A**. For simulation, a full human single-cell profile (origin profile) is defined by a full bulk profile and the corresponding sparse single-cell profile is defined by the down-sampled bulk profile. Compared to real data, the simulations allows us to test SIMPA on full profiles related to a large variety of ChIP protein targets and biosamples. Using the origin profile as the validation set of true binding interactions, the area under ROC-curve (AUROC in y-axis) describes the capability of SIMPA to accurately impute the sparse profile and recapitulate the origin. The bars describe the mean AUROC and the error bars describe the standard deviation across multiple applications on sparse sets with different sizes. SIMPA was validated with two strategies, the default leave-out origin (LOO; origin profile excluded from the training set) and the extreme leave-out cell-type (LOCT; all experiments with the same cell type than the origin profile are excluded from the training set). The x-axis labels indicate the cell-type of the origin profile and additionally the ENCODE accession to show which of the experimental dataset was used as origin. **B**. Same as in **A** but using the area under precision-recall curve (AUPRC in y-axis) as performance measure. The pink bars show the class balance (fraction of positives in the class feature) representing the random assumption as baseline to be expected from a primitive classifier that randomly assigns the class values (according to [29]). Note, the sampled bins, that simulate a sparse single-cell profile and are expected to be known before imputation, were completely excluded before computing area under the curve values.

For H3K4me3, the most frequently investigated target in ENCODE, high area under ROC-curve values confirm that SIMPA is able to accurately recapitulate the original data from the simulated sparse profiles (Fig 2A). Even if the cell-type-specific information is completely removed from the training set (LOCT), the performance is still high. Furthermore, these observations are confirmed when using precision-recall curves as performance measure (Fig 2B), a relevant analysis given the imbalance in the validation sets (containing far fewer positive than negative samples). We made similar observations in a ROC-curve and precision-recall curve analysis for other cell-type-target combinations (**Figs S4 and S5 in** S1 File).

**Fig 3:**
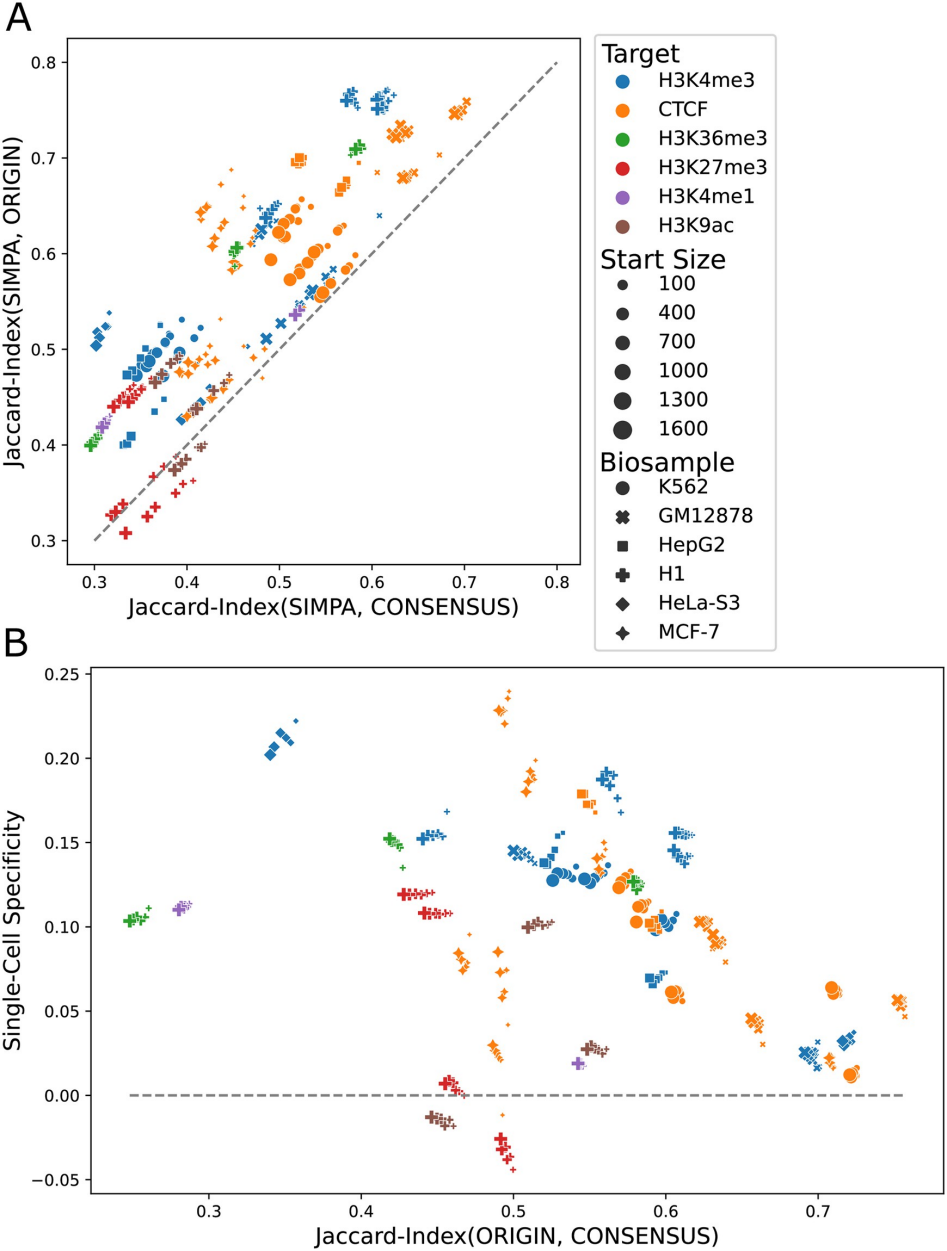
Single-cell specificity analysis. **A**. The Jaccard-Index is used to compare the imputed profiles from SIMPA with the origin human bulk profile used to create a simulated sparse profile and the consensus profile representing the remaining experiments available for the same biosample-target combination as the origin profile. The dashed line shows the balance line at which the imputed profile from SIMPA is neither closer to the origin nor to the consensus. Cases above the dashed line are those in which the imputed profile is single-cell specific, hence, closer to the origin than to the consensus. **B**. “Single-Cell Specificity” on the y-axis is defined as the difference between the imputed-to-origin similarity (y-axis in **A**) and imputed-to-consensus similarity (x-axis in **A**). Having the similarity between the origin and the consensus on the x-axis, this plot allows the visualization of the single-cell specificity in relation to how specific the origin is. The higher the similarity between the origin and consensus, the less specific is the origin profile and the harder the challenge to capture its specificity. Profiles above the 0 line are single-cell specific as they are closer to the origin than to the consensus. Before computing the Jaccard-Index values, the sampled bins, which simulate a sparse single-cell profile and are expected to be known before imputation, were removed from all the sets, origin, imputed, and consensus.

In order to assess the single-cell specificity of SIMPA in this simulation, we compared each fully imputed profile to its origin profile and also to a consensus profile representing experimental datasets that are most similar to the origin (experimental profiles with same protein target and same cell-type, see **Supplementary Note 3 in** S1 File for details). All comparisons excluded bins used as input for SIMPA. Results show that for most of the simulations (>95%) the imputed profile is closer to the origin profile, hence single-cell specific (Fig 3A). Moreover, we observed that the origin profiles can be more similar to the consensus profile (less specific) or less similar (more specific). When the origin profiles are less specific, it is harder for SIMPA to achieve an imputed profile specific to the origin (single-cell specific). However, for such cases in which the origin is quite close to the consensus (Jaccard-Index > 0.65) the imputation is still single-cell specific, although with a lower single-cell specificity value (Fig 3B).

Taken together, the simulation results show that models trained from a few bins accurately impute thousands of bins and show that completed profiles can be single-cell specific on real data even if the investigated cell-type is not represented by any of the bulk datasets in the reference set (leave-out cell-type validation).

### Model interpretability on real data

Addressing one main aim of this study to make models interpretable, we implemented an extension called InterSIMPA. Here we define interpretability as the possibility of obtaining information of potential biological relevance from the relationships observed between the training features (genomic bins) and a genomic position of interest. These relations can be expected to be part of the genomic regulatory network.

The training features are derived by InterSIMPA in the same way as for SIMPA but a single machine learning model is trained for a selected genomic position of interest defined by the user. Accordingly, one imputed probability is returned for genomic position of interest with information about the genomic bins most important for the machine learning model. Finally, the algorithm reports the genes closest to these bins (**Supplementary Note 4 in** S1 File).

To demonstrate how interpretable imputation models can be used to expose more information from the sparse ChIP-seq profile of individual single cells, we use the human single-cell ChIP-seq dataset of H3K4me3 interactions in B-cells and T-cells from Grosselin *et al*. [4]. According to the given cell types, we focused on promoter regions of genes that are involved within the B-cell and T-cell receptor signaling pathways. The two gene sets contain 67 and 97 genes for the B-cell receptor and T-cell receptor signaling pathways, respectively, with an overlap of 44 genes. To focus on the genes that could be more specific to the cell-types under investigation, from the union of the two gene sets we selected 24 genes with frequency of their promoter regions lower than 20% in the corresponding H3K4me3-specific reference set, which means that their promoter has no detected interaction site for more than 80% of the ENCODE reference experiments for H3K4me3 in different cell-types and tissues (**Supplementary Note 4 in** S1 File).

**Fig 4:**
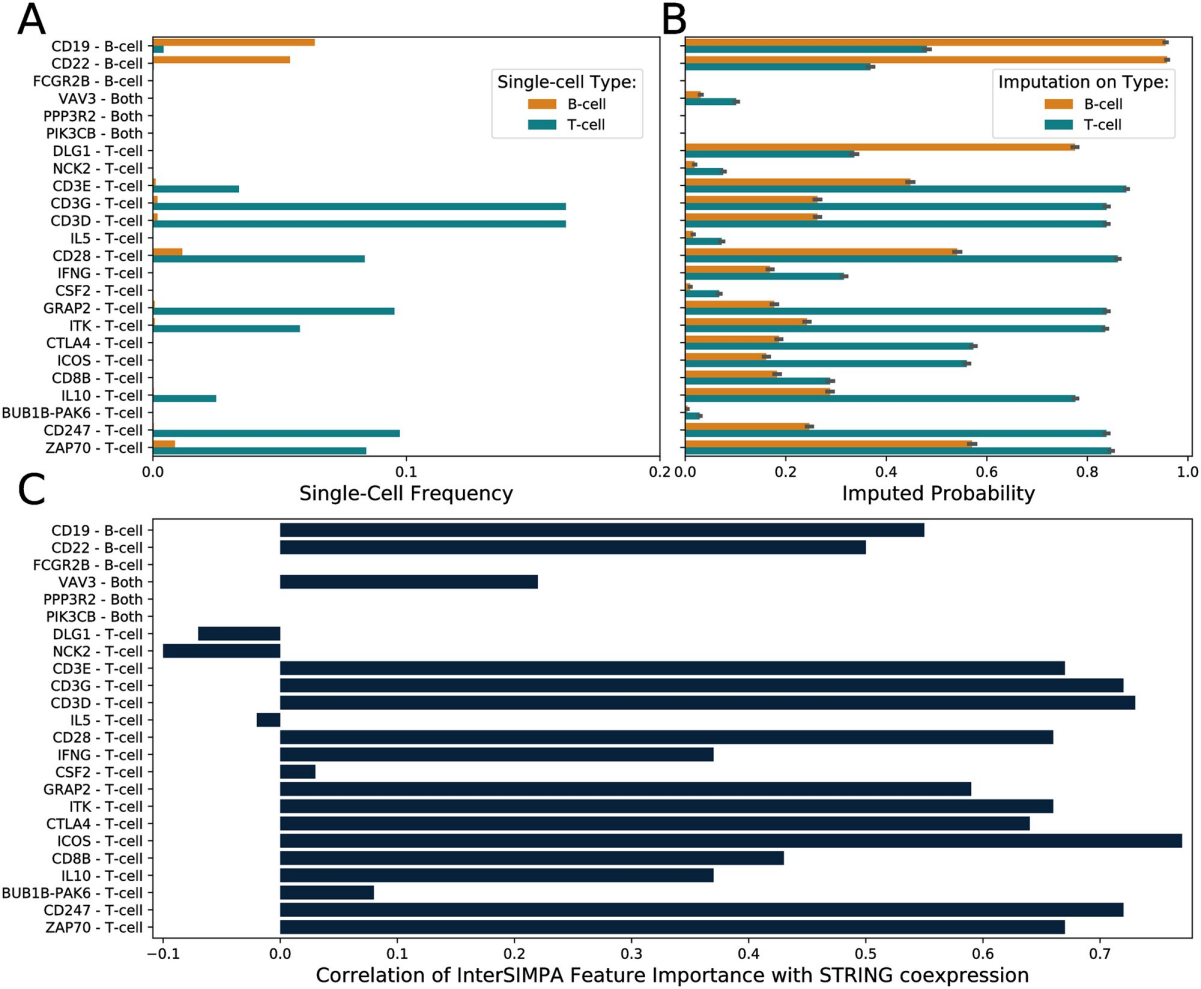
Pathway related gene analysis using the interpretation of imputation models. ** A**. Fraction of single cells for which H3K4me3 binding is observed within the gene’s promoter region in the human single-cell dataset (orange and blue bars representing B-cells or T-cells, respectively). Y-axis labels show the gene names and if the gene belongs to the B-cell or T-cell receptor signaling pathway or to both. **B**. Imputed probability computed by SIMPA for the gene-related promoter regions shown in A. The imputation was applied on numerous single cells from the cell-types B-cell (orange) and T-cell (blue). The error bars represent the standard deviation across the imputation runs on different cells. For the majority of genes, the imputed probability is higher within the cell-type that corresponds to the gene’s pathway. **C**. Correlation of feature importance and co-expression values. For each model used to impute a promoter (y-axis), the training features (genomic bins) were extracted together with their importance value provided by the Random Forest algorithm and annotated with the nearest gene on the genome. Co-expression values, derived from transcriptomic and proteomic measurements, of those genes with the gene related to the imputed promoter were retrieved from the STRING database. The Pearson correlation coefficient of feature importance and co-expression values is shown (x-axis).

As H3K4me3 is an activating histone mark, we expected to observe interaction sites in the promoter regions of these genes. However, for many of those promoter regions, the H3K4me3 binding is missing for most of the single cells in the sparse data (Fig 4A). Our expectation that SIMPA is able to impute such regions at least in a cell-type-specific manner, is confirmed by comparing the imputed probabilities calculated by SIMPA for promoter regions of the 24 selected genes in single B- and T-cells (Fig 4B). For most of the genes, the imputed probability is higher when SIMPA is applied on single cells that are from the pathway-related cell-type. Finally, we evaluated the interpretability of the 24 imputation models by comparing feature importance values and co-expression values of the feature-related genes with the gene of the imputed promoter (Fig 4C). Co-expression data from the STRING database was used [30]. The observed high correlations suggest that InterSIMPA is capable to describe biologically relevant promoter-promoter relations by the predictive information hidden within sparse histone mark profiles of an activating mark. Consequently, our approach not only completes the sparse scChIP-seq dataset, but its interpretability-extension is even capable of providing deeper insights into the data.

### Performance on cell-type clustering and functional analysis

After the evaluation of the InterSIMPA extension, here, we evaluate how SIMPA enhances single-cell data corresponding to different cell types. For the imputation of a full single-cell dataset, SIMPA was applied for each cell individually. The resulting imputed profiles were then analyzed within two validations, to examine if (i) cell-type clustering was retained after imputation and (ii) if the imputed single-cell profiles are significantly associated with genes of the corresponding cell-type-specific pathway. Following the investigations of Schreiber *et al*. [31], we also compared bin probabilities from SIMPA to a simple imputation approach that uses bin frequencies in the reference set (experiments with same protein target) as a probabilistic model without using any machine learning model, called the *average interaction* method. Additional imputations and randomization tests were applied and compared to better analyze the basic concept of SIMPA (see **Supplementary Note 5 in** S1 File).

**Fig 5:**
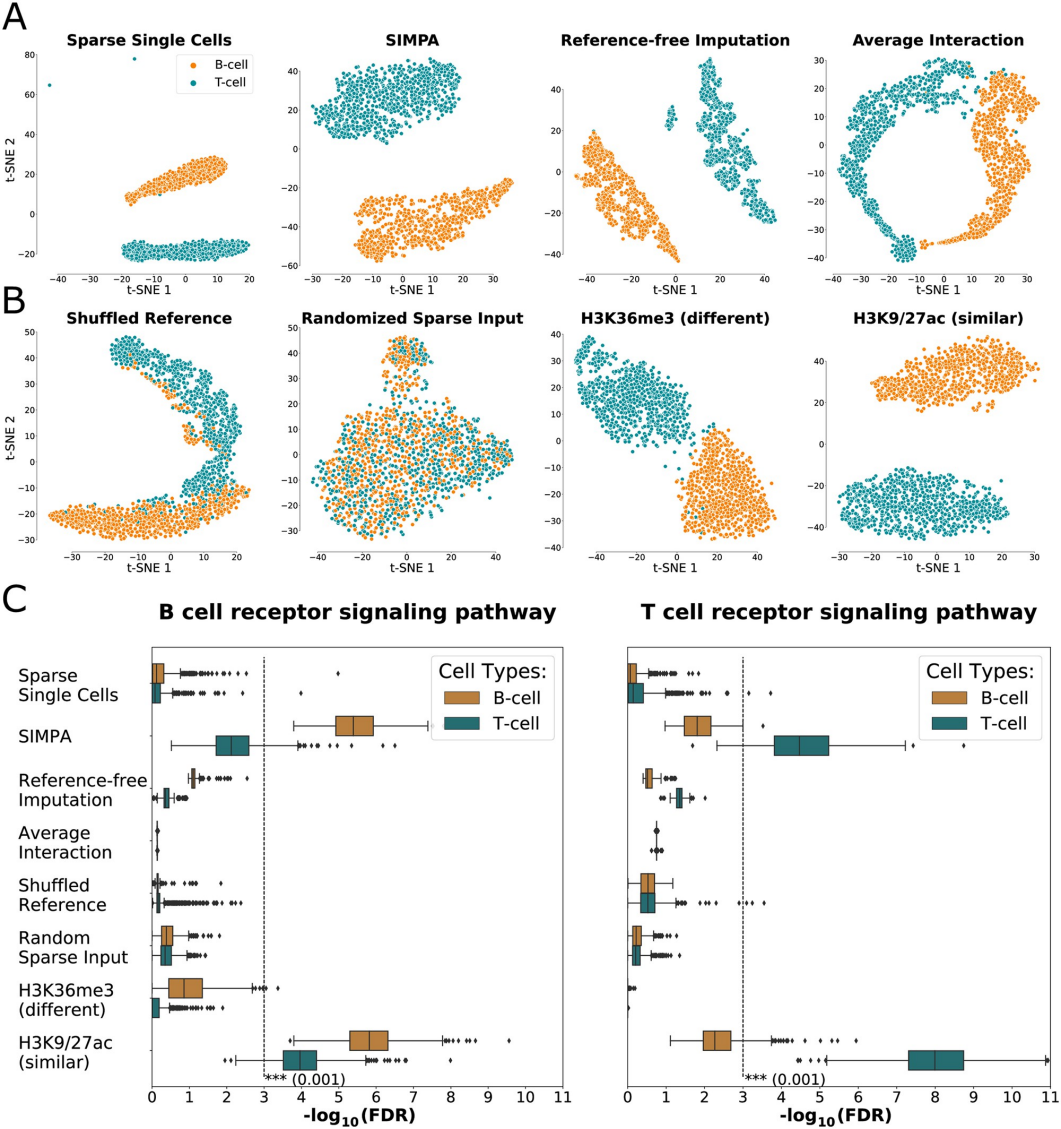
Cell-type specificity validation. **A + B Separation of single cells according to cell type**. **A**. Dimensionality reduction analysis applied on the human H3K4me3 data derived from (i) the sparse single-cell data and three different imputation methods, (ii) SIMPA, (iii) reference-free imputation, and (iv) average interaction based on expected frequencies in the reference set. Results from SIMPA and from the reference-free method achieve the best clustering by separating the single cells (points) by cell types (colors). **B**. Effects of input modification on SIMPA, (i) using a shuffled reference set or (ii) randomized sparse input data, or using other histone marks as reference instead of H3K4me3, either (iii) the functionally different histone mark H3K36me3, or (iv) the functionally similar histone marks H3K9ac and H3K27ac. Only SIMPA used with relevant protein targets was able to correctly cluster all single cells (no outliers). **C. Pathway enrichment analysis**. Boxplots show the significance of pathway enrichment analyses of genes annotated by single-cell regions as log-transformed false discovery rate (FDR; x-axis). Each dot represents the FDR of one single cell from the results of the different analysis experiments shown in **A+B** (y-axis). The dashed lines represent the log-transformed significance threshold of an FDR equal to 0.001. Only SIMPA achieves significant results by imputing preferably genomic regions associated with relevant pathway-related genes.

Because of the better resolution available for H3K4me3 processed as genomic bins of size 5 kb in the human dataset, we present below results on this histone mark and refer to supplementary material for H3K27me3 processed at 50 kb bins (**Fig S6 in** S1 File). For benchmarking, we used a single-cell ATAC-seq imputation method, SCALE, solely based on the single-cell dataset itself (*reference-free*) in contrast to SIMPA, which takes advantage of information from the reference bulk dataset. After applying a two-dimensional projection on the sparse and imputed datasets, we observed that the separation between the cell types was retained by SIMPA and by the reference-free method, contrary to the average interaction method (Fig 5A). Moreover, three T-cell outliers were successfully associated to the related cell-type cluster by SIMPA, which achieved a slightly better homogeneity of the clusters in comparison to SCALE which did not handled correctly the outliers (**Fig S6 in** S1 File). Dimensionality reduction was done by a combination of principal component analysis (PCA) and t-stochastic neighbor embedding (t-SNE) as suggested by Grosselin *et al*. on their analysis of sparse data [4]. Unlike the suggested procedure, we did not perform cell filtering, as we were interested to observe outliers after imputation.

In order to validate further the algorithmic concept of SIMPA, we implemented two randomization tests in which either the ENCODE reference information was shuffled (Shuffled Reference) or the sparse single-cell input was randomly sampled (Randomized Sparse Input). Additionally, we applied SIMPA on the same data but with models trained for different related or unrelated histone marks. The selected histone marks were H3K36me3, a histone mark functionally different to H3K4me3, and H3K9ac and H3K27ac, a group of two histone marks functionally related to H3K4me3. These two marks were used together to increase the training data size. From this comparison, we observed that (i) the separation on the projection is lost after removing statistical patterns through shuffling or randomization, (ii) separation quality is moderate with an input mark functionally different from the real mark, and (iii) separation quality stays high using SIMPA with target histone marks functionally similar to the real mark (Fig 5B). Thus, the most relevant statistical patterns from the reference dataset are identified by both the selection of single-cell-specific regions and the selection of target-specific experiments. Similar observations were made for H3K27me3 although a more compact clustering could be achieved on the SIMPA profiles compared to those from the reference-free method (**Fig S6 in** S1 File). Across several dimensionality reduction procedures applied for the H3K4me3 dataset, SIMPA and the reference-free method were both stable in retaining the cell-type clustering (**Fig S7 in** S1 File). From these analyses we additionally conclude that the UMAP method using the Jaccard-Index distance achieves reasonable results when applied directly on the sparse data (in comparison to the common approach in single-cell analysis that uses first a PCA to select dimensions).

As pathway enrichment analysis is a common step in ChIP-seq data exploration, we next investigated if enrichment analyses of cell-type-specific pathways for individual single cells improve after applying imputation. We analyzed the sparse profiles and different imputed results with the KEGG pathway analysis function of the Cistrome-GO tool [32]. As reported in Fig 5C, the original sparse data did not provide enough interaction sites to show a significant pathway enrichment for any of the two cell types. Results from the reference-free strategy showed an improvement but not significant. However, with regions imputed by SIMPA, it was indeed possible to achieve significant enrichment scores and recover the cell-type-specific pathways for most of the cells. These results show that SIMPA is able to integrate functionally relevant information from the reference data in order to impute additional biologically meaningful bins, in contrast to the reference-free method, which is limited to the single-cell dataset.

### Optimal size of the imputation sets

As described, SIMPA computes the imputed probabilities for numerous genomic bins, and sorts and prioritizes those accordingly for imputation. In the previous validations, as a default, we imputed a number of bins equivalent to the average number of bins observed across all bulk ChIP-seq profiles from the target-specific reference set. On 5 kb resolution, the average number of bins of the H3K4me3 experiments is 32,584. However, once the bins are ranked by the imputed probability it is up to the user to alternatively create imputed sets of different sizes. With the next analysis we address the question about the optimal number of bins needed to improve the cell-type clustering at the same time enabling the detection of the relevant biological function by a significant enrichment of the correct pathway (for details see **Supplementary Note 6 in** S1 File).

**Fig 6:**
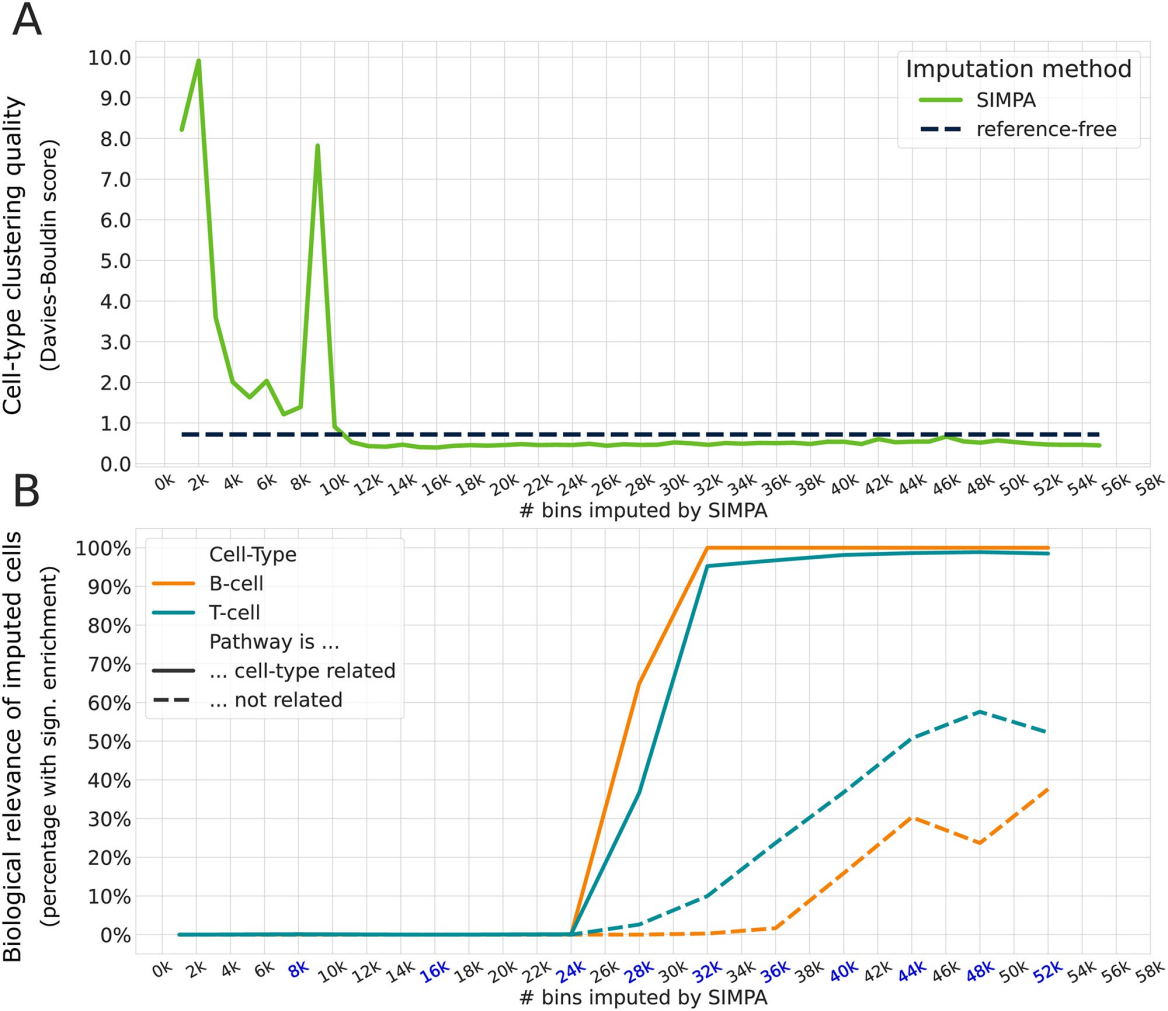
Clustering quality and pathway enrichment for different sizes. **A**. Clustering quality (y-axis) evaluated with the Davies-Bouldin score (the lower the better) applied on the imputed data after dimensionality reduction as described for Fig 5A+5B. While the reference-free method derived only one imputed set for all the single cells (dashed black line), we could derive several imputed sets of different sizes using the imputed probabilities from SIMPA (x-axis). **B**. The pathways under investigation are the B-cell and T-cell receptor signaling pathways. In this way we analyze two pathways surely related or unrelated with the cell-types present in the dataset, B-cell and T-cell. The y-axis describes the percentage of imputed profiles for which a significant enrichment of the aforementioned pathways could be achieved. The dashed lines represent cases for which the unrelated pathway is significantly enriched, which is the T-cell receptor signaling pathway when analyzing a B-cell and vice versa. The significance level used is 0.001 similar to the analysis shown in Fig 5C. To reduce the computational resources spent on the pathway enrichment, this was done for ten imputation set sizes (highlighted in blue on the x-axis).

The best cell-type clustering quality, evaluated by the Davies-Bouldin score [33], is reached when adding ~11,000 bins (Fig 6A). At this level, SIMPA slightly improves the clustering quality compared to the reference-free method.

Considering the amount of cells in which the cell-type related pathway is significantly enriched, we observed that in ~50% of the cells the related pathway is associated when adding ~28,000 bins (Fig 6B). After adding more than 32,000 bins, almost all cells have a significant enrichment for the cell-type related pathway, however, it seems to be also the limit for avoiding the association of the unrelated pathway. For this analysis the same pathways and settings are used as in Fig 5C; the unrelated pathway is the T-cell receptor signaling pathway when analyzing a B-cell and vice versa.

### InterSIMPA applied on mouse scChIP-seq data (Zhu et al., 2021)

**Fig 7:**
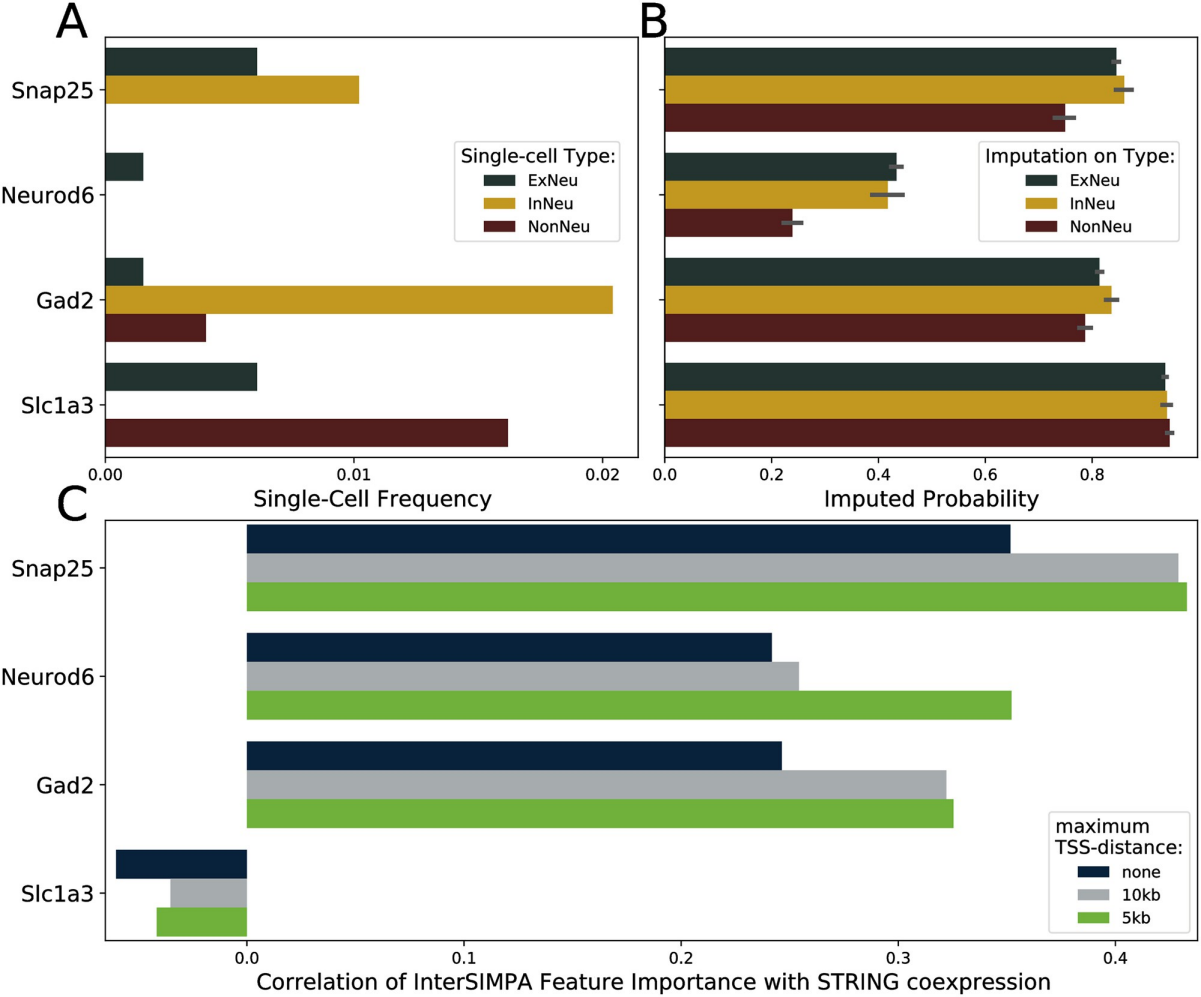
Gene analysis using the interpretation of imputation models. **A**. Fraction of single cells for which H3K4me3 binding is observed within the gene’s promoter region in the mouse single-cell dataset. Dark green and yellow bars representing excitatory (ExNeu) and inhibitory (InNeu) neurons, respectively. Red bars represent non-neurons (NonNeu). y-axis labels show the gene names. **B**. Imputed probability computed by SIMPA for the gene-related promoter regions shown in A. The imputation was applied on 1000 single cells in total and the error bars represent the standard deviation across the imputation runs on different cells. **C**. Correlation of feature importance and co-expression values. For each model used to impute a promoter (y-axis), the training features (genomic bins) were extracted together with their importance value provided by the Random Forest algorithm and annotated with the nearest gene on the genome. As indicated by the figure legend, the selection of genomic bins was either not restricted (none), or restricted by a maximum distance to the transcription start site (TSS) of the nearest gene. Co-expression values, derived from transcriptomic and proteomic measurements of those genes with the gene related to the imputed promoter were retrieved from the STRING database. The Pearson’s correlation coefficient of feature importance and co-expression values is shown (x-axis).

New technologies allow to obtain the joint profiling of histone modifications and transcriptome in single cells as used by [24]. From this dataset we chose H3K4me3 profiles from mouse brain cells available on 1kb binning resolution to further validate the concept of InterSIMPA. As each single-cell dataset is very specific to the investigated cell-types, we focused on four genes also used within the original study to analyze the difference between excitatory and inhibitory neurons, as well as non-neurons. We first annotated the promoter regions of these genes based on 1000 randomly selected sparse single-cell profiles and observed a very low coverage of the promoter regions ranging from 0.0 to ~2.0% (Fig 7A). Despite the low single-cell coverage, the patterns agree with those from the original study (see Fig 1F in [24]); note that we focus on the promoter only and do not consider the full gene body. We then analyzed the imputed probabilities from InterSIMPA for these four genes (Fig 7B). For the three genes *Snap25*, *Neurod6*, and *Gad2*, the imputed probability is higher for the neurons compared with the non-neurons, and no difference can be observed between cell types for *Slc1a3*. Similarly for *Snap25*, *Neurod6*, and *Gad2*, a moderate positive correlation could be observed between feature importance values and STRING co-expression data, but not for *Slc1a3* (Fig 7C). By using the appropriate parameter of the tool, we restricted the InterSIMPA output to regions within the proximity of 10kb or 5kb, respectively. As shown in Fig 7C this has a positive impact on the results from InterSIMPA as it increased substantially the correlation calculated for the three genes.

## Discussion

After confirming the presence of statistical patterns within the ENCODE bulk ChIP-seq reference data, we show that machine learning models can leverage those patterns for the accurate inference of interaction sites in sparse single-cell ChIP-seq profiles from individual single cells.

The investigation of protein-DNA interactions on single-cell resolution emerged more recently compared to gene expression (single-cell RNA-seq) or chromatin accessibility (single-cell ATAC-seq) and consequently, less datasets are available for scChIP-seq. The human dataset we chose for our analysis includes profiles for H3K4me3 in 5kb resolution and H3K27me3 in 50kb resolution in human B-cells and T-cells. This dataset is appropriate as it provides a clear cell-type annotation which enabled an analysis based on the pathways we would expect to be assigned to B-cells and T-cells, respectively. Contrary to the human dataset from Grosselin *et al*., we observed that a very complex procedure was applied in the study presenting the mouse dataset from Zhu *et al*. to reveal the different cell clusters [24]. From this study we chose the only profile available on a higher resolution (1kb), which describes the histone mark H3K4me3. Using common dimension reduction methods for single-cell datasets, we were able to reproduce clustering results for the Grosselin dataset (Fig S7 in S1 File) but it was not possible to recapitulate the mouse cell types, neither before nor after imputation (results not shown). This could be explained by the complexity of the joint profiling of different histone marks within single cells and by the fact that the Zhu and colleagues could not apply a barcoding strategy to annotate the cell-types as it was done for the Grosselin dataset. Therefore, we extensively used the human dataset together with simulations to validate SIMPA and limited the use of the mouse dataset to the validation of the model interpretability offered by the InterSIMPA extension.

Based on the simulations, we could show that the imputed results obtained by SIMPA are single-cell-specific for several cell-type-target combinations even if the experiments related to the cell-type were completely excluded from the training set. In both types of validation (leave-out origin and leave-out cell-type validations), SIMPA was able to capture cell-type-specific patterns even though the reference set was composed of profiles from many different cell-types and tissues, or the cell-type related data was completely excluded. Because the number of available bulk experimental profiles (ENCODE datasets) differs between targets, different training set sizes are available for different targets, with the smallest training set for H3K9ac (49 biosamples). Even for training sets of smaller size, the predictive performance remained high, although we expect models to be more reliable the larger the training set. Given that data portals such as ENCODE are still growing, we expect that the model reliability will increase in the future for many targets with a growing number of available reference datasets.

The interpretation of the SIMPA models, done with InterSIMPA applied on a real scChIP-seq dataset published by Grosselin and colleagues, allows us to reveal additional information from the ChIP-seq profiles measured within individual cells regarding regions responsible for the imputation. Importantly, leveraging reference data allows us to impute regions that were not present in the single-cell dataset at all, in contrast to a reference-free strategy. Considering for example the promoter regions of the T-cell receptor signaling pathway genes *CTLA4* and *ICOS*, these promoter-regions are not detected in any of the cells from the Grosselin *et al*. dataset, however, both have a high imputed probability from SIMPA. Moreover, for both promoters a high correlation coefficient was achieved within the validation by STRING co-expression values, confirming that our implementation not only answers the question about whether these promoters should be imputed or not, but it additionally reveals valuable information about regulatory relations implied by the single-cell dataset.

Regarding the full data imputation analysis, we observed further advantages of SIMPA’s reference-based imputation strategy compared to the reference-free imputation method. While both algorithms achieve a good separation of the cell types, only with the imputed profiles from SIMPA it was possible to determine the relevant biological function of single cells as shown by the pathway enrichment analysis. This suggests that SIMPA imputes biologically meaningful genomic bins which are of functional relevance and confirms that, even though the training set involves a variety of different tissues and cell-types, SIMPA can find statistical patterns that belong to the correct cell-type. For single-cell datasets which reveal unknown subpopulations of cells, SIMPA could be used to identify active pathways for those cells after imputation. Interestingly, the quality of those results was maintained to some extent when not exactly the same scChIP-seq histone-mark target but functionally related targets were used to define the reference set. This suggests a valuable strategy to be applied for targets with a low availability of public bulk reference profiles.

Given the results achieved by InterSIMPA on H3K4me3 single-cell profiles for mouse brain cells, we see a difference in performance compared to those achieved on human B-cells and T-cells that might be explained by the more complex cell type identification in the original study from Zhu *et al*. [24]. However, we still see higher imputed probabilities of *Snap25*, *Neurod6* and *Gad2* in excitatory and inhibitory neurons compared to non-neurons, which confirms the relevance of those genes to a cell-type-specific study. This could not be observed for *Slc1a3*. The validation analysis based on the STRING co-expression data also highlighted the potential involvement of the three genes in regulatory networks. This analysis, which is also integrated within InterSIMPA, provides information about the reliability of the imputed probabilities. The gene *Slc1a3* serves as an example for which the non-specificity of the imputed probabilities agrees with the low correlation coefficient between the InterSIMPA feature importance and STRING co-expression.

SIMPA integrates solely datasets from bulk ChIP-seq in order to build the reference set. However, in the future, it will be relevant to integrate other types of data in order to complementarily extend the reference set. For example, SCRAT is an analysis tool that summarizes single-cell regulome data using different types of public datasets such as genome annotations or motif databases that could be of interest for the application of SIMPA to transcription factor scChIP-seq profiles [34]. The scATAC-seq analysis tool SCATE performs imputation of missing regions integrating different types of public datasets (e.g. co-activated cis-regulatory elements and bulk DNase-seq profiles) [35]. For future work, such approaches suggest the development of a reference-based method, allowing the imputation for both scChIP-seq and scATAC-seq data, integrating both types of reference data from the corresponding bulk assays and further complementary datasets.

In the current version, SIMPA and InterSIMPA use all bulk profiles as selected by the target(s) specified by the user. Yet, there is no parameter that allows a user to further restrict the reference set by cell-types or tissues. We expect that this is not necessary as the machine learning algorithm can find the relevant patterns in the training set due to the additional specification induced by the given single-cell profile, which provides enough information about its cell-type despite its sparsity. Especially the pathway enrichment analysis supports this assumption since the imputed regions are related to the true biological function of the given cell-types. Nevertheless, for a future extension of the algorithm, it might be beneficial to investigate the impact of further specifying the reference set by tissues or cell-types related to the single-cell dataset.

We demonstrate that the usage of bulk reference data can be beneficial in scChIP-seq imputation, especially when the single-cell data set leaves genomic regions uncovered. Given a single cell as input, its profile is used to specify the underlying training set to receive a result individual for the cell. Thus, single-cell and bulk information are combined in the imputation concept we propose. However, the imputation might improve for one individual cell by incorporating information from other, maybe similar, cells from the single-cell dataset. Recently, it was shown by the method scAND that the concept of network diffusion can successfully be applied in scATAC-seq imputation [36]. In this study a bipartite network is created in which the edges describe if a region is accessible in a cell; no bulk reference is involved in this concept. However, considering the integration of bulk data, it might be possible to expand such a network by patterns detected in a bulk reference set. Edges could then describe different states for genomic regions depending on their occurrence in the single-cell and bulk datasets. Such networks would describe a complex composition of information and concepts from multi-graph theory might be helpful to extract patterns from these networks relevant for imputation [37].

SIMPA’s strategy, to train a model for each candidate bin and each single cell, results in its capability to produce highly relevant results and at the same time in its main limitation which is the requirement of a large amount of computational resources. Using a high-performance cluster, the results presented in this manuscript could be obtained within 1–2 days. However, if computational resources are limited, SIMPA offers the opportunity to run the imputation for a selection of cells which, for instance, represent a certain cluster to be analyzed. As shown, cell clusters can be identified even on the sparse profiles using the appropriate method for dimensionality reduction. Importantly, InterSIMPA can also be applied for individual cells, providing interpretable results within seconds of runtime.

On two datasets we demonstrated the potential of the concept of involving bulk data especially for ChIP-seq imputation, a topic less covered by other studies so far. The application of the proposed methods is possible as we provide the source code with comprehensive explanations in a github repository. However, our study might additionally serve as a guideline to be considered for the development of new imputation methods. As applied in the study from Zhu and colleagues [24], it is possible to profile several histone marks and the transcriptome of single cells simultaneously and we expect that such techniques will be further improved in the near future. The resulting new datasets are expected to come up with a high level of sparsity, and imputation methods will be needed; though, there will be new requirements for imputation methods as single cells are then described by a variety of profiles describing a variety of biological functions. The complexity of information available for each single cell should be included in a novel imputation method applicable for such datasets. We therefore expect that future steps in single-cell imputation will involve the development of methods able to incorporate several protein-DNA interaction profiles and the transcriptome of single cells to integrate all these data for more robust imputation results. Based on the findings of this study we suggest to consider the integration of corresponding bulk data as well. Moreover, interpretability concepts for the imputation models should also be included within the development of future methods as it reveals detailed insights into the single-cell dataset under investigation.

## Conclusion

The strategy of SIMPA leveraging bulk ChIP-seq datasets for single-cell sequencing data imputation, is able to complete specifically sparse scChIP-seq data of individual single cells. In comparison to the non-imputed data and a reference-free imputation method, SIMPA was better at recovering cell-type-specific pathways. Furthermore, the interpretability of the machine learning models trained for the imputation can be used to reveal biologically important information from a sparse single-cell dataset. Conclusively, we developed an ensemble of computational methods to extract more information from a sparse dataset and impute missing data to better handle data sparsity of scChIP-seq datasets.

## Supporting information

S1 File Contains supplementary notes 1 to 7 and supplementary figures S1 to S7. (DOCX)

S1 Table (XLSX)
